# Academic resilience and academic performance of university students: the mediating role of teacher support

**DOI:** 10.3389/fpsyg.2025.1463643

**Published:** 2025-04-30

**Authors:** Zimo Cai, Qian Meng

**Affiliations:** College of Education, Bohai University, Jinzhou, China

**Keywords:** academic resilience, teacher support, academic performance, correlation analysis, mediation analysis

## Abstract

**Introduction:**

Student dropout and academic failure are serious problems faced by universities around the world. The academic development of university students is the joint result of external (teacher support) and internal factors (resilience). The goal of this study is an in-depth understanding of the association between university Students’ academic resilience and academic performance.

**Methods:**

All respondents were from a local Chinese public university. Two self-report scales with adequate reliability and validity were completed by 440 undergraduate students: the Connor-Davidson Resilience Scale (CD-RISC) and the Perceived Teacher Support Scale (PTSS).

**Results:**

The results of this study demonstrated that academic resilience and teacher support were positively and closely correlated with the academic performance of university students. Teacher support plays a mediating role in the nexus between academic resilience and academic performance.

**Discussion:**

The findings of this study shed light on the improvement of university students’ academic performance and reducing the dropout rate from theoretically and practically. The study helps identify the key factors that contribute to students’ academic success and improve their well-being. The results also provide evidence for university directors to design effective programs and take initiatives to increase retention rates and improve university students’ success.

## Introduction

Student dropout and academic failure have become serious problems faced by universities around the world, in the United States (ThinkImpact, 2023), Europe (Behr et al., 2020), Spain (Ortiz-Lozano et al., 2018), and Indonesia (Najimi et al., 2013; Nurmalitasari et al., 2023). The United States has an alarming 40% college dropout rate per year, with only 41% of students successfully completing their 4-year degrees (ThinkImpact, 2023). According to a survey by the education website, Intelligent.com, 28% of college students did not return for the fall 2022 semester because of poor academic performance (National Student Clearinghouse Research Center, 2022). Universities in China encounter similar challenges and issues, including the need for increased accountability, efficiency and quality, financial difficulties, market pressures, and the expansion and diversification of their student bodies (Hong, 2018). This issue is especially severe in science and engineering courses, where the failure rate of students can reach up to two-thirds. Even at top schools, such as Tsinghua and Zhejiang Universities, fifth- and sixth-year engineering students find that they are unable to complete their courses (Zhang, 2019).

Previous studies have explored the determinants of academic performance, including demographic (gender, age), individual (social background, past performance), psychological (motivation, attitudes), institutional (teaching quality, learning environment) and national (financing policy) determinants (Alyahyan and Düştegör, 2020). Of the individual determinants, resilience is an important positive psychological factor in explaining academic performance (Salanova et al., 2009). Academic resilience referred to a capacity to overcome acute and/or chronic adversity that is seen as a major threat to a student’s educational development(Martin and Marsh, 2009; Martin, 2013). Previous studies have shown that academic resilience can positively affect academic performance (Ayala and Manzano, 2018; Cassidy, 2016; Meneghel et al., 2019). A four-week longitudinal study involving 74 Chinese college students found that resilience could enhance their academic performance (Li et al., 2024). Therefore, research on the psychological resilience of college students is conducive to improving their academic performance, which, in turn, helps reduce the dropout rate.

Another determinant related to the academic performance of university students is teacher support. Previous research has yielded widely recognized findings, primarily grounded in data gathered from classrooms in the United States and western countries. These findings emphasize that teachers are the most crucial resource within the educational system, playing a pivotal role in fostering student learning and development (Affuso et al., 2023; Sabol et al., 2013). The study, which recruited 593 university students in Guizhou Province, China, demonstrated that when teachers provide more support and resources, they can enhance students’ academic self-efficacy and positive academic emotions, thereby ensuring students’ sustained engagement and optimal academic performance (Ma et al., 2023). According to the self-determination approach, teacher support occurs when students perceive cognitive, emotional, or autonomy-oriented support from a teacher during their learning process (Lei et al., 2018).

Existing research has provided a good foundation for verifying the relationship between academic resilience, teacher support, and academic performance. However, most previous research studies have only discussed the direct relationship between academic resilience, teacher support and academic performance (Ayala and Manzano, 2018; Mercer et al., 2011), but have ignored the possible mediating role of teacher support at university level. A recent study showed that students with higher levels of resilience perceived and recruited teachers’ support to enhance their engagement (Romano et al., 2021). There is a need for more in-depth research on the relationship between academic resilience and academic performance through the mediating role of teacher support.

This study attempts to investigate the relationship between academic resilience and academic performance in the Chinese university context. Meanwhile, the mediating role of teacher support will be examined. All the respondents were from a local public Chinese university. This study adds to the body of literature examining the link between the academic resilience and the academic performance of university students; it attempts to identify the mediating function of teacher support, an aspect which was overlooked in the other studies. This study also highlights the importance of expounding the interrelationships of academic resilience, teacher support, and academic performance in different demographic groups. Furthermore, this study sheds light on how to reduce dropout rates and increase students’ academic performance by enhancing students’ academic resilience.

## Literature review and hypothesis development

### Academic resilience and academic performance

Morales and Trotman described academic resilience as the process and outcomes that are part of the life story of a person who has succeeded academically despite challenges that prohibit the majority of people with similar backgrounds from accomplishing their goals (Morales and Trotman, 2004). The definition of academic resilience is based entirely on achieving exceptional academic success despite facing challenges or hardships. Academic resilience has been identified as a significant characteristic of all students who face severe adversities during their academic careers (Ayala and Manzano, 2018). Academic resilience consists of the following three constructs: tenacity, strength, and optimism. The tenacity dimension encapsulates an individual’s composure, prompt response, persistence, and sense of mastery when confronted with difficult and challenging situations. The strength dimension indicates an individual’s capacity to bounce back from setbacks. The optimism dimension assesses how an individual perceives the positive elements within various situations (Song et al., 2021).

Academically resilient students reported higher academic performance and more engagement in studying than their counterparts (Allan et al., 2014). A four-week longitudinal survey involving 74 Chinese college students confirmed that resilience has a positive impact on college students’ well-being and mental health (Li et al., 2024). A survey of 206 students indicated that there are strong, clear associations between better academic performance and higher resilience (Egan et al., 2022). A research of 68 collegiate students revealed that students who develop resilience are more likely to sustain high levels of achievement, motivation, and performance despite the presence of stressful conditions (Carsone et al., 2024; Ye et al., 2024). However, there is currently no consensus on the relationship between academic resilience and academic performance. Some studies have demonstrated that there is no (or a weak) association between academic resilience and academic performance (Beauvais et al., 2014; Elizondo-Omaña et al., 2010). Based on previous studies, we propose the following hypothesis:

*H1*: Academic resilience is positively related to university students’ academic performance.

### Teacher support and academic performance

According to ecological system theory (Bronfenbrenner, 1977), one of the most significant and direct microsystems that affects learning outcomes is a teacher-supported classroom setting. Teachers have a protective function in students’ development and are one of the most important sources of social support for young students (Quin et al., 2018). For adolescents who are experiencing uncertainty or trauma, it may be especially important to develop strong, dependable relationships with their teachers. Teacher support is a promising factor in reducing stress and improving students’ academic performance (Hoferichter et al., 2022).

Teacher support, as an important social support, has been regarded as an important indicator of promoting students’ academic performance (Vargas-Madriz and Konishi, 2021). Teacher support is a complex concept that incorporates the following three dimensions: structure, autonomy support, and involvement. Structure refers to the quantity of information in the context of how to effectively attain desired results; the opposite of structure is chaos. The amount of freedom a student is given to choose his or her own behavior is referred to as autonomy support; coercion is the reverse of support. Involvement describes how well students get along with their teachers and peers; its antithesis is rejection or neglect (Skinner and Belmont, 1993).

A survey of 419 adolescents over 3 years showed that teacher support directly and positively affected motivation and self-efficacy over time, which, in turn, positively impacted academic performance (Affuso et al., 2023). A survey involving 298 Chinese undergraduate students showed that teacher support exerts a tremendous influence on boosting Chinese students’ academic engagement (Pan and Yao, 2023). Teacher support is associated with students’ academic self-concept and academic enjoyment, which, in turn, improve students’ performance (Ma et al., 2021). However, according to Mercer et al.’s study, there is no correlation between academic improvement and teacher support over the course of the academic year (Mercer et al., 2011). The relationship between teacher support and student academic performance merits further research. In view of the above conflicting results, we propose the following hypothesis:

*H2*: Teacher support is positively associated with university students’ academic performance.

### The mediating role of teacher support

Students’ academic development is the complex result of internal–personal and external–environmental factors. These factors can be classified into either internal–personal or external–environmental categories (Dwiastuti et al., 2021). Internal–personal factors are those that are controlled inside the individual in response to different situations; examples include self-efficacy (Dogan, 2015), motivation (Meng and Hu, 2023), resilience (Ayala and Manzano, 2018) and self-set goals (Alessandri et al., 2020). External–environmental factors refer to factors existing outside individuals and that have an impact on their academic success; examples include institutions (Sandoval-Hernández and Bialowolski, 2016), families (Agasisti and Longobardi, 2017), teachers (Mercer et al., 2011), and peers (Bronfenbrenner, 1977). A research study conducted in Spain showed partial mediation of academic support in the relationship between students’ personal resources and academic engagement (Robayo-Tamayo et al., 2020). More specifically, resilient students perceive and utilize teachers’ support to increase their engagement. A study by Lobo verified that the higher the resilience level of students, the more they can perceive higher emotional support from their instructors (Lobo, 2023).

Previous studies indicate the value of expanding the research on the mediating effect of teacher support between academic resilience and academic performance. Based on the above elucidation, we propose the following hypothesis:

*H3*: Teacher support will positively mediate the relationship between academic resilience and academic performance.

### Conceptual framework

A conceptual framework is a representation of the relationship between variables and provides a roadmap for conducting research. Conceptual frameworks describe a broad overview of the subject and outline key concepts, variables, and the relationships between them (Kahu, 2013). Social Cognitive Theory, proposed by Albert Bandura in the 1960s, is a psychological theory that emphasizes the reciprocal interactions among individuals, environment and behaviors. Social cognitive theory is a valuable theory for investigating academic performance among university students (Honicke et al., 2023; Ye et al., 2024). Psychological resilience, as a personal factor and teacher support, as an environmental factor, jointly affect students’ academic performance.

Previous research has proved the importance of academic resilience and teacher support in improving academic performance and reducing dropout rates. The purpose of this study is to clarify how academic resilience combined with teacher support influence the academic performance of university students. The framework is intended to be helpful in directing future research and designing interventions aimed at enhancing students’ academic performance. However, the majority of existing studies consider the direct correlation between academic resilience, teacher support, and academic performance separately; the potential mediating function of teacher support between academic resilience and academic performance has been neglected. More research is required to improve our comprehension of the various factors that affect academic achievement and to develop programs to enhance students’ academic performance and personal growth, ultimately helping to prevent student dropout.

**Figure 1:**
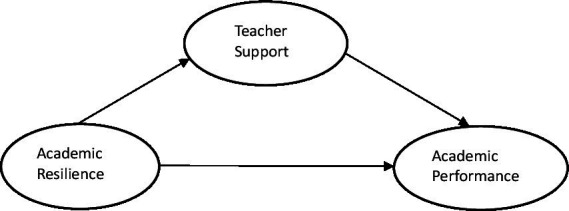
The estimated model.

This study seeks to investigate the nexus between academic resilience and the academic performance of university students in greater detail. The effect of academic resilience on academic performance was investigated by testing teacher support as a potential intermediary variable. The meditating role of teacher support is underpinned by the presumption that students who possess greater personal resources tend to seek out additional external resources to help them achieve success (Lorente et al., 2014). In accordance with this evidence, the intermediate role of teacher support is acknowledged. Therefore, this study attempts to examine the nexus between academic resilience and academic performance by analyzing the mediation function of teacher support. The estimated model is shown in Figure 1.

## Methodology

### Sample

All participants in this study were students enrolled in a Chinese public university with the goal of enhancing students’ academic performance and improving the university’s ranking. We used a simple random sampling method to select individuals from undergraduate students. All undergraduate students had an equal probability of being selected. This method tends to produce representative, unbiased samples (Mitra and Pathak, 1984). Students were approached during scheduled classes or seminar sessions. We took a multi-channel approach to distributing the questionnaires, including online and offline, depending on the preferences and accessibility of our target respondents to ensure the validity and reliability of our findings. All participants were active full-time undergraduate students. Exclusion criteria involved students enrolled in overseas and part-time programs. This is because this study focuses on examining the correlation between academic resilience, teacher support, and academic achievement in a Chinese setting and provides a clearer and more nuanced understanding of this particular subset of the university population.

All the respondents participated voluntarily in the study and were given written consent forms to ensure that they voluntarily agreed to take part in the study and to adhere to its ethical regulations (according to the Declaration of Helsinki and later amendments). The respondents consisted of 163 males (37%) and 277 females (63%). Gender imbalance should be understood through representativeness and statistical power (Dickinson et al., 2012). The gender distribution of the sample aligns with that of the students at this comprehensive university, which primarily specializes in the liberal arts. The apparent gender imbalance is a statistical representation of the student population The participants were from social humanities (40.7%), natural science (26.6%), and engineering (32.7%). Regarding class standing, 15.2% were freshmen, 23.9% were sophomores, 32% were juniors, and 28.9% were seniors. Table 1 shows the demographic breakdown of categorical factors (gender, class rank, and program).

**Table 1:** Demographic breakdown of categorical variables.

Variable	*n*	%
Gender		
Male	163	37
Female	277	63
Class standing		
Freshman	67	15.2
Sophomore	105	23.9
Junior	141	32
Senior	127	28.9
Program		
Social humanity	179	40.7
Natural science	117	26.6
Engineering	144	32.7

### Procedure

The study was conducted from March 2023 to June 2023 with participants randomly recruited from a university in China. We clearly stated the purpose of the questionnaire and assured the participants of the confidentiality of their responses. Prior to participating, the individuals were given a verbal overview of the research objectives and provided with relevant information to ensure a thorough understanding of the project details. They were requested to complete the CD-RISC and PTSS scales and informed that they had the right to withdraw from the study if they felt uncomfortable. They were also advised that they could refuse to answer any specific question and that none of the participants would be named in any publication. Every effort was made to protect their identities. At the end of the questionnaire, each participants had the option to provide their email address if they wished to receive a summary of the study results. These measures effectively ensured the rationality and integrity of the data collected while simultaneously safeguarding the privacy and rights of the participants.

### Data analysis

In the data analysis section, the Shapiro–Wilk test was used to verify the normal distribution of the data, indicating that the data conforms to a normal distribution (*p* > 0.1). In the process of data processing and cleaning, the missing values were supplemented with the mean value. Meanwhile, Harmon’s single factor test was used to test for common method bias, and the results showed that the explanatory power of the first factor did not exceed the critical value of 40%, indicating that there was no common method bias in the data (Fuller et al., 2016). This study used regression analysis and mediation tests to verify the impact of psychological resilience on academic performance and the mediating role of teacher support and to explore the relationship between them.

### Measures

All the respondents completed two self-report scales with sufficient reliability and validity, the CD-RISC (Connor and Davidson, 2003) and the PTSS. Yu developed the Chinese version of the CD-RISC questionnaire, designed by Connor and Davidson in 2007 (Yu and Zhang, 2007). The Chinese version of the CD-RISC questionnaire had good reliability and validity in the Chinese context in a survey of 976 college students (Chen et al., 2020). The CD-RISC scale consists of three dimensions: tenacity (13 items, e.g., Even in the face of despair, I remain persistent and do not succumb to defeat.), strength (8 items; I always strive to give my best effort, regardless of the circumstances.), and optimism (4 items, I can deal with whatever comes). Respondents were instructed to respond to a 5-point Likert scale, from 1 (not true at all) to 5 (true all the time). The higher the score, the higher the level of academic resilience. The internal consistency coefficient for the CD-RISC scale was 0.90. Confirmatory factor analysis was used to test the model fit, and the fitting indices met the criteria (RMSEA = 0.054, IFI = 0.91, TLI = 0.90, CFI = 0.92, GFI = 0.91).

The average variance extracted (AVE) for tenacity, strength, and optimism was 0.77, 0.76, and 0.75, respectively, and the composite reliability (CR) for tenacity, strength, and optimism was 0.82, 0.85, and 0.83, respectively. In line with to Fornell and Larcker, the AVE value more than or equal to 0.5 confirms convergent validity and the benchmark value of higher than 0.7 indicates good internal consistency reliability (Fornell and Larcker, 1981). As Table 2 shows, there is a significant correlation between the three dimensions; the absolute value of the correlation coefficient is less than 0.5 and less than the square root of the corresponding AVE, indicating that the scale has adequate discriminant validity.

**Table 2:** Discriminant validity and correlation among variables of academic resilience scale.

Variables	Strength	Tenacity	Optimism
Strength	0.873^a^		
Tenacity	0.426***	0.879^a^	
Optimism	0.335***	0.368***	0.869^a^

***p < 0.001; ^a^Diagonals represents the square root of average variance extracted, whereas the rest of the entries represent the latent variable correlations.

Perceived teacher support in relation to students’ academic performance was assessed using a 19-item PTSS scale. The Chinese version of the PTSS was revised by Ouyang based on Babad’s study (Babad, 1990), and the reliability and validity of the PTSS have been well verified in a study involving 740 adolescents (Zeng et al., 2022). It includes the following three dimensions: learning support (9 items, e.g., When I am unable to answer a question, the teacher will explain the question repeatedly to me), emotional support (6 items, e.g., My teacher often encourages me in my studies and daily life), and capacity support (4 items, e.g., My teacher has consistently encouraged my involvement in a variety of activities and competitions.) The questionnaire uses Likert 5-point scoring (1 = strongly disagree, 2 = disagree, 3 = not sure, 4 = agree, 5 = strongly agree); the higher the questionnaire score, the more teacher support students perceived. The Cronbach’s *α* coefficients of the PTSS scale were 0.89, and 0.85, 0.82, 0.83 for learning support, emotional support, and capacity support, respectively. The outcomes of the confirmatory factor analysis provided evidence that all the fit indices were within acceptable bounds (RMSEA = 0.067, IFI = 0.91, TLI = 0.90, CFI = 0.91, GFI = 0.93). This confirms that the PTSS has good structural validity in China.

The convergent validity (AVE) for learning support, emotional support, and capacity support were 063, 0.62, 0.65, respectively, and the CR for learning support, emotional support, and capacity support were 0.86, 0.83, and 0.84, respectively. As Table 3 shows, the three dimensions of the PTSS were significantly correlated, and the absolute value of the correlation coefficient was much less than the square root of the corresponding AVE, thereby satisfying the criteria for adequate structure validity.

**Table 3:** Discriminant validity and correlation among variables of teacher support scale.

Variables	Learning support	Emotional support	Capacity support
Learning support	0.794^a^		
Emotional support	0.347***	0.790^a^	
Capacity support	0.419***	0.324***	0.811^a^

***p < 0.001; ^a^Diagonals represents the square root of average variance extracted, whereas the rest of the entries represent the latent variable correlations.

The cumulative grade point average (GPA) of the students who were enrolled in the university up to the date of the study was employed to evaluate academic performance of university students. Students reported their own GPA. The GPA value went from 1 (poor) to 5 (excellent) according to the credit system used in higher education in China.

## Results

### Descriptive statistics and correlation analyses

In this survey, we handed out 500 questionnaires, 440 of which were completed, representing 88% of the total. Table 4 shows the results of the descriptive and correlation coefficients analyses. The findings showed a substantial positive link between academic resilience, teacher support, and academic performance in the university student population. The correlation coefficient between academic resilience, teacher support, and academic performance was 0.63 (*p* < 0.001) and 0.61 (*p* < 0.001), respectively. Additionally, the correlation coefficient between academic resilience and teacher support was 0.64 (*p* < 0.001). Therefore, to ascertain the relationship between academic resilience and academic performance, further investigation of the mediating effect of teacher support was needed.

**Table 4:** Correlation coefficients between academic resilience, teacher support and academic performance.

Variables	*M*	SD	1	2	3
1 Resilience	3.25	0.75	1		
2 Teacher support	3.25	0.80	0.636***	1	
3 Academic achievement	3.02	0.60	0.608***	0.643***	1

***p < 0.001.

As Table 5 shows, the results revealed significant gender difference in CD-RISC score (*t* = −5.23, *p* < 0.001). Females score higher than males on the psychological resilience scale because they are generally more expressive and communicative about their emotions, which helps them process and cope with stress (Chaplin, 2015). However, there was no significant difference in the PTSS scale. The findings also revealed significant differences in scores for the two scales by class standing (*F* = −63.03, *p* < 0.001; *F* = 34.92, *p* < 0.001) and program (*F* = 44.98, *p* < 0.001; *F* = 31.15, *p* < 0.001). Addressing the potential implications of gender, class standing, and program differences in resilience scores is vital for fostering a personalized and supportive environment across diverse contexts.

**Table 5:** Group differences in academic resilience and teacher support among university students.

Variable	Academic resilience	Teacher support
Gender		
Male (163)	2.69 ± 0.78	2.90 ± 0.36
Female (277)	3.04 ± 0.68	2.91 ± 0.51
*t*	−5.23***	0.012
Class standing		
Freshman (67)	3.14 ± 0.16	2.77 ± 0.18
Sophomore (105)	2.51 ± 0.16	2.58 ± 0.12
Junior (141)	3.43 ± 0.08	3.18 ± 0.10
Senior (127)	2.62 ± 0.11	3.21 ± 0.14
*F*	63.03***	34.92***
Program		
Social humanity (179)	2.87 ± 0.26	2.73 ± 0.19
Natural science (117)	3.51 ± 0.18	3.22 ± 0.23
Engineering (144)	2.69 ± 0.14	3.02 ± 0.17
*F*	44.98***	31.15***

### Mediation analyses

Under the control of gender, class standing, and program, academic resilience had a significant direct predictive effect on academic performance (*β* = 0.49, *t* = 16.01, *p* < 0.001), supporting H1. Even when the mediating variable (teacher support) was entered into the regression link, the indirect effect of academic resilience on academic performance remained quite significant (*β* = 0.27, *t* = 7.59, *p* < 0.01), supporting H3. As Table 6 shows, academic resilience was significantly positively correlated with teacher support (*β* = 0.64, *t* = 16.43, *p* < 0.001). Teacher support was a robust predictor of academic achievement, with a meaningful positive impact (*β* = 0.35, *t* = 10.46, *p* < 0.01), supporting H2.

**Table 6:** Mediating effect test between academic resilience, teacher support, and academic performance.

Variable	Academic performance		Academic performance		Teacher support	
	*β*	*t*	*β*	*t*	*β*	*t*
Gender	0.196	3.517**	0.096	1.568	−0.284	−3.626**
Class standing	0.052	2.009*	0.018	0.640	−0.095	−2.612**
Program	−0.021	−0.601	−0.012	−0.321	0.024	0.484
Academic resilience	0.265	7.590***	0.490	16.013***	0.642	16.426***
Teacher support	0.352	10.455***				
*R* ^2^	0.502		0.376		0.430	
*F*	87.370***		65.565***		81.919***	

The intermediary role of teacher support in the relationship between academic resilience and academic performance was examined using a PROCESSOR macro developed by Hayes (2013). The PROCESS macro is the most commonly used approach to evaluate mediation effects in the field of psychology, and in other fields. In small sample research, a bootstrap resampling method has been shown to be more effective in testing mediation effects than a large sample method (Alfons et al., 2018). Meanwhile, SEM was further used to verify the mediating role of teacher support. In the field of education and psychology, SEM have shown unique advantages, especially analyzing the impact of one variable on another through a third variable (MacCallum and Austin, 2000; Zhang et al., 2024).

**Figure 2:**
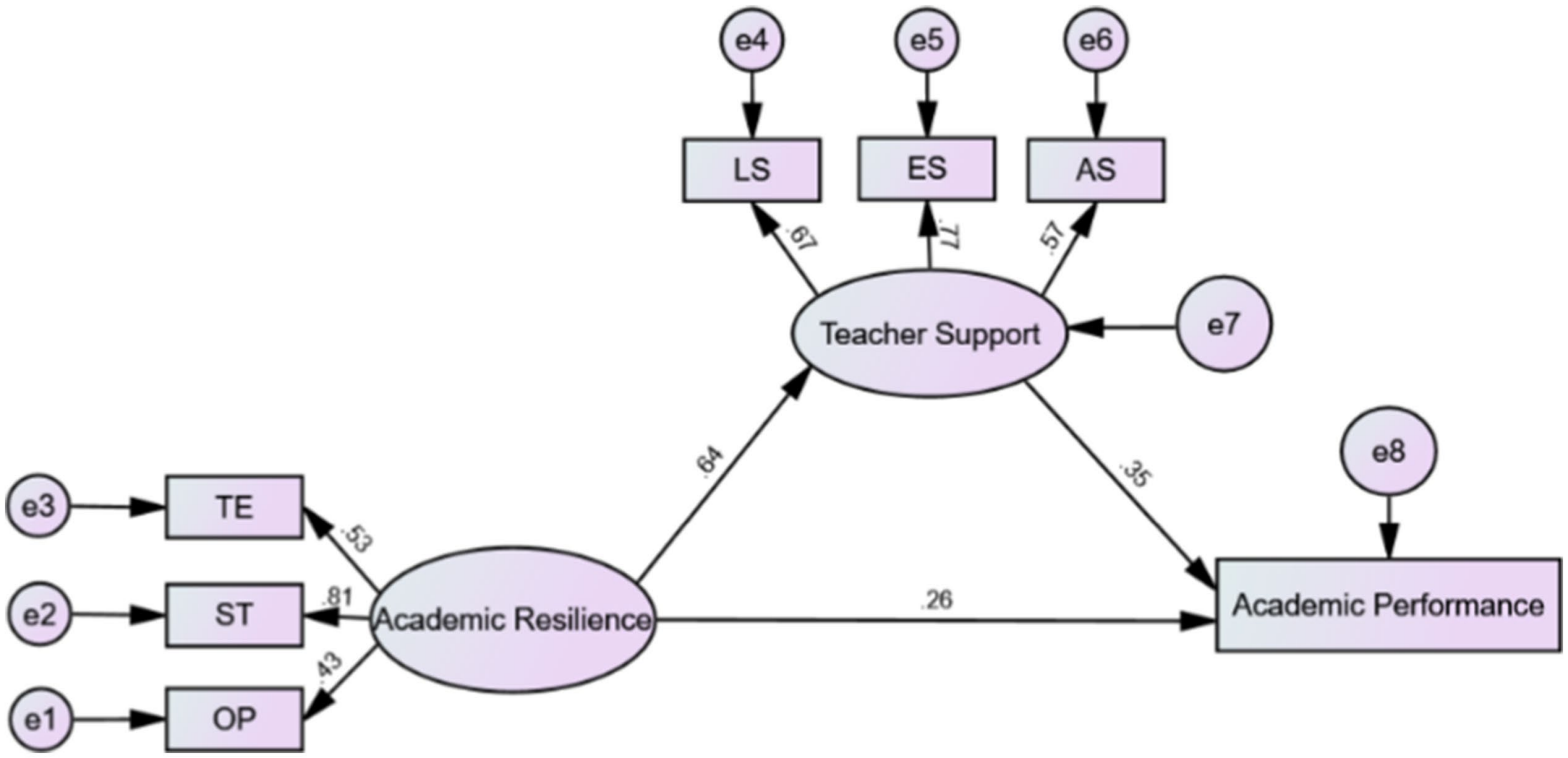
Mediating model of academic resilience, teacher support, and academic performance.

The significance of direct, indirect, and total effects in the mediation analysis model was tested after eliminating the demographic factors (gender, class standing, and program), see Table 7. The 95% confidence interval (CI) of the direct impact of academic resilience on academic performance was [0.20, 0.33], with a value of 0.26 constituting 53.99% of the total effect. Moreover, the indirect impact value of academic resilience on academic performance mediated by the influence of teacher support (95% bootstrap, CI = 0.17, 0.29) was 0.23, accounting for 46.01% of the total effect. The findings indicate that academic resilience has a direct link with academic performance and an indirect impact on academic performance through the mediating influence of teacher support. Figure 2 shows the specific path of the impact of academic resilience on academic performance and the mediation path of teacher support.

**Table 7:** The test of total effect, direct effect, and indirect effect.

Effect	Effect	BootSE	BootLLCI	BootULCI	Relative effect value
Total	0.4903	0.0306	0.4301	0.5504	
Direct effect	0.2647	0.0349	0.1962	0.3332	53.99%
Indirect effect	0.2256	0.03	0.1663	0.285	46.01%

## Discussion

The aim of this study was to investigate the correlation between academic resilience, teacher support, and academic achievement in a sample of students from a public university. Correlation analysis showed that academic resilience was significantly positively correlated with the academic performance of university students. Mediation analysis showed that teacher support plays a mediating role in the nexus between academic resilience and academic performance. The results supported H1, H2, and H3 as showed in Table 8.

**Table 8:** The results of hypothesis testing.

Hypotheses	Results
H1: Academic resilience is positively related to university students’ academic performance	Supported
H2: Teacher support is positively associated with university students’ academic performance	Supported
H3: Teacher support will positively mediate the relationship between academic resilience and academic performance	Supported

First, the results support H1, which is consistent with other research studies and shows a direct and significant relationship between academic resilience and the academic achievement of university students (*r* = 0.608, *p* < 0.01; *β* = 0.49, *t* = 16.01, *p* < 0.001) (Ayala and Manzano, 2018; Cassidy, 2016; Meneghel et al., 2019). Students with high levels of resilience tend to perform better academically due to their persistence, determination, and self-efficacy. Academic resilience is often referred to as “better than expected” educational outcomes (Borman et al., 2004). A student with strong academic resilience is better equipped to cope with the challenges and stressors of higher education, such as workload, assigned tasks, and exams. Moreover, resilient students tend to possess better self-regulation skills, persistence, and goal-setting abilities, vital components for success in academia. Academic resilience can help predict the academic performance of university students.

Second, the findings of this study support H2 by showing that teacher support had a favorable impact on university students’ academic performance, consistent with earlier studies (*r* = 0.643, *p* < 0.01; *β* = 0.35, *t* = 10.46, *p* < 0.01) (Hoferichter et al., 2022; Vargas-Madriz and Konishi, 2021). Given the favorable and encouraging association between teacher support and students’ academic performance, we are certain that teacher support improves students’ learning outcomes, especially in high-stakes testing and accountability contexts (Tao et al., 2022). Teacher support has a significant impact on academic performance as it can motivate, guide, and empower students to succeed academically. When teachers support their students, they show their concern and care for them, and students frequently express their respect for the teacher by upholding the classroom rules (Longobardi et al., 2016). Students tend to feel more secure and believed when their teachers provide supportive and positive feedback. This relationship can foster open communication and mutual respect, making the learning environment a more comfortable one, leading to better performance.

Finally, the findings of this study support H3 by showing that teacher support acts significantly as a mediating variable between the academic resilience and academic success of university students (*β* = 0.27, *t* = 7.59, *p* < 0.01). A study showed that students who perceived their teachers’ support were more motivated and involved in their studies than their counterparts (Reyes et al., 2012). In line with previous studies, students who display greater resilience compared to their peers tend to report greater levels of perceived social support and a more favorable perception of their learning environment (Romano et al., 2021). Therefore, resilient students have a greater willingness to establish a reliable and supportive relationship with their teachers, who are key to helping students bounce back from difficulties, thereby improving their academic performance. Teachers who provide a supportive and caring environment for their students can help foster resilience in them, providing them with essential resources and tools to cope with academic challenges.

### Implications

The findings of this study shed light on improving university students’ academic performance and reducing the dropout rate both theoretically and practically. Academic resilience is a vital aspect of academic success. It enables students to adapt to challenges and setbacks while still maintaining academic performance (Cassidy, 2016). There are many factors that can affect the psychological resilience of university students. These include burnout (Pahwa and Khan, 2022), social support, and relationships with family or friends (Auttama et al., 2021). In these areas, teacher support plays a pivotal role in enhancing students’ psychological resilience. High-quality educator–learner relationships offer a support base for students’ learning in the long term. Students in a classroom with a supportive teacher are more likely to have higher engagement and performance (Tao et al., 2022). When students feel supported by their teachers, they tend to be more motivated to learn and do well in class. This study confirms that teacher support can be an essential factor in mediating the relationship between academic resilience and academic performance. The fostering of a positive and inclusive learning environment, clear and achievable expectations, effective leveraging of technology, and the establishment of positive teacher-student relationships, will enable educators to create an engaging classroom experience that cultivates resilience, helping students to overcome academic challenges and achieve success in their studies (Affuso et al., 2023; Maria, 2021).

Teacher training programs should focus on enhancing teachers’ understanding and support for students’ academic resilience; they should cultivate teachers’ positive feedback capacity and strengthen their support and counseling skills (Pozo-Rico et al., 2023). Teachers should create a supportive and interactive classroom environment to encourage students to become more resilient in their academic pursuits. Policymakers and practitioners should provide high-quality training to teachers to enable them to provide meaningful academic support to students.

Where institutional policies are concerned, research indicates that students facing the greatest academic challenges could benefit most from supplemental instruction and tutoring services (Balzer and London, 2019). One study found that 60% of university students reported clinical levels of stress (Stallman, 2016). These tutoring programs can vary significantly, ranging from those offering peer counseling or advising those arranging for expert instructors to supplement course instruction. Collaboration between faculty members, administrative staff, and student support services can also create a more cohesive and effective support system for students.

### Limitations and future directions

While the research on academic resilience, teacher support, and academic performance offers important insights into educational practice and policy, there are some limitations to this study that should be considered. First, the participants in this study all come from a Chinese public university, and, while this has little bearing on the research findings, it might limit the generalizability of the findings to other demographics. It would be interesting in further studies to test the results in other universities or other populations. By conducting replication studies, researchers can assess the consistency of findings across different contexts and establish the reliability and validity of the results. Second, this study employed a cross-sectional approach to establish the association between academic resilience, teacher support, and academic performance. This approach does not allow for definitive conclusions about causality. Cross-sectional studies cannot establish causation, as they provide a snapshot of variables at a single point in time (Savitz and Wellenius, 2023). Hence, longitudinal research is encouraged to provide stronger evidence to identify the causal relationships between variables. A key advantage of longitudinal research is that it can establish the temporal order of variables. This is crucial for identifying causality because it allows researchers to determine whether one variable (e.g., academic resilience, teacher support) precedes and potentially influences another variable (e.g., academic performance) (Ployhart and Vandenberg, 2010). In addition, university students’ academic performance is influenced by various internal and external factors that go beyond academic resilience and teacher support. These include personal factors such as motivation, self-esteem, and anxiety, as well as social factors such as family background, economic status, and cultural expectations (Alabdulkarem et al., 2021; Behr et al., 2020; García, 2021; Najimi et al., 2013). It is challenging to examine all these factors in one research project, as they may involve different theoretical constructs and methodologies. Further research could discuss these variables in greater depth.

## Conclusion

This study aims to investigate how academic resilience influences academic performance, as well as the mediating effect of teacher support. The results indicate a favorable correlation between academic resilience, teacher support and academic performance. The findings also confirm the mediating effect of teacher support on the nexus between academic resilience and academic performance. The novelty of the study lies precisely in its emphasis on the mediating role of teacher support between academic resilience and academic performance. By highlighting the mediating effect of teacher support, our study seeks to fill a gap in the existing literature. The findings suggest that teacher support is not just a peripheral factor, but rather a critical component that can influence how students’ resilience leads to academic outcome.

For educators, the findings of this study show that support from teachers is a crucial factor in determining the academic achievement of students who display resilience. This finding adds to the understanding of how resilience and teacher support can positively impact university students’ abilities to succeed academically, even in the face of challenges. Specifically, educators should prioritize building strong supportive relationships with students. By creating a supportive environment at the classroom level, teachers can improve students’ academic performance and foster resilience, contributing positively to student retention. Educators should also offer tailored academic guidance, addressing individual student needs and challenges to support students’ success.

For institutions, the results highlight that academic resilience is a crucial personal trait for academic success. Universities should recognize the importance of cultivating students’ academic resilience and implement programs to promote students’ academic resilience. It is crucial for universities to train teachers, ensuring they are equipped with skills and knowledge to provide effective support. Universities should also establish comprehensive student support systems that integrate various forms of assistance, from academic counseling to resource provision.

The China focus on academic resilience, teacher support and academic performance of university students aligns well with global trends, as educational institutions worldwide are increasingly recognizing the importance of fostering resilience in students (Carsone et al., 2024). This study helps identify the key factors that contribute to students’ academic success and improve their well-being. The results also provide evidence for university directors to design effective programs and take initiatives to increase retention rates and improve university students’ success. These findings can be generalized to other cultural and educational settings, promoting a more nuanced understanding of how to support students globally.

In conclusion, academic resilience and teacher support both play an integral role in determining the academic performance of university students, and a combination of these factors can increase greater success in higher education.

## Data Availability

The raw data supporting the conclusions of this article will be made available by the authors, without undue reservation.
